# 橙皮苷诱导慢性髓性白血病细胞系K562细胞发生铁死亡的作用及分子机制

**DOI:** 10.3760/cma.j.cn121090-20231218-00323

**Published:** 2024-06

**Authors:** 俊毅 魏, 龙 李, 慧敏 刘

**Affiliations:** 1 山西医科大学第二医院血液科，太原 030001 Department of Hematology, the Second Hospital of Shanxi Medical University, Taiyuan 030001, China; 2 山西医科大学细胞生理学教育部重点实验室，太原 030001 Key Laboratory of Cellular Physiology（Shanxi Medical University）, Ministry of Education, Taiyuan 030001, China

**Keywords:** 白血病，髓系，慢性, 橙皮苷, 铁死亡, SLC7A11/GPX4轴, Leukemia, myeloid, chronic, Hesperadin, Ferroptosis, SLC7A11/GPX4 axis

## Abstract

**目的:**

探讨橙皮苷诱导慢性髓性白血病细胞系K562细胞发生铁死亡的作用及分子机制。

**方法:**

通过CCK-8、EDU-594、Transwell法检测橙皮苷对K562细胞活力、增殖和迁移的影响。采用流式细胞术检测K562细胞的凋亡率。采用C11-BODIPY和FerroOrange检测细胞中脂质过氧化和Fe^2+^水平。通过Western blot法检测细胞中铁死亡相关蛋白溶质载体家族7成员11（SLC7A11）、谷胱甘肽过氧化物酶4（GPX4）表达水平。采用SLC7A11过表达质粒转染细胞后，同样检测脂质过氧化及Fe^2+^水平。

**结果:**

橙皮苷可呈剂量依赖性降低K562细胞活力，IC_50_值为0.544 µmol/L，选取0.4、0.8 µmol/L的橙皮苷行后续实验。EDU-594、Transwell法和流式细胞术检测显示，0.4、0.8 µmol/L橙皮苷作用24 h后K562细胞增殖和迁移率明显降低，细胞凋亡率明显提高，与对照组比较差异均有统计学意义（*P*值均<0.05）。同时Western blot法检测显示，抗凋亡蛋白Bcl-2表达下调，促凋亡蛋白Bax及Caspase-3表达升高。与对照组比较，橙皮苷可以提高细胞内脂质过氧化和Fe^2+^水平（*P*值均<0.05）。铁死亡抑制剂（Fer-1）与橙皮苷联合给药可以逆转橙皮苷对K562细胞的作用。0.8 µmol/L橙皮苷作用组铁死亡相关基因SLC7A11、GPX4 mRNA及蛋白水平明显降低（*P*值均<0.05）。SLC7A11过表达可以抑制橙皮苷作用，减轻铁死亡。

**结论:**

橙皮苷可通过调控SLC7A11/GPX4轴，促进K562细胞铁死亡。

## Abstract (en)

**Objective:**

To investigate the effect and molecular mechanism of hesperadin in inducing ferroptosis in chronic myeloid leukemia cell line K562 cells.

**Methods:**

The effects of hesperadin on the viability, proliferation, and migration of K562 cells were detected though CCK8, EDU-594, and Transwell assays, and the apoptotic rate of K562 cells was detected by flow cytometry. In addition, C11-BODIPY and FerroOrange were utilized to detect intracellular lipid peroxidation and Fe^2+^ levels. Meanwhile, the expression levels of ferroptosis-associated protein solute carrier family 7 member 11（SLC7A11）and glutathione peroxidase 4（GPX4）in cells were detected through Western blot. Lipid peroxidation and Fe^2+^ levels were also detected after transfection of cells with SLC7A11 overexpression plasmid.

**Results:**

Hesperadin decreased cell viability in a dose-dependent manner with IC_50_ of 0.544 µmol/L. Hesperadin concentrations of 0.4 and 0.8 µmol/L were selected for follow-up experiments. EDU-594, Transwell, and flow cytometry showed significantly decreased proliferation and migration rate of K562 cells after 0.4 and 0.8 µmol/L hesperadin treatment for 24 h, and the apoptosis rate was significantly increased compared with the control group（*P*<0.05）. Western blot indicated a downregulated expression of the antiapoptotic protein Bcl-2 and an elevated expression of proapoptotic proteins Bax and Caspase-3. Moreover, hesperadin increased intracellular lipid peroxidation and Fe^2+^ levels compared with the control treatment（*P*<0.05）. The combination of ferroptosis inhibitor（Fer-1）and hesperadin could reverse the effect of hesperadin on K562 cells. The mRNA and protein levels of ferroptosis-related genes SLC7A11 and GPX4 were significantly decreased in the 0.8 µmol/L hesperadin-treated group（*P*<0.05）. SLC7A11 overexpression can inhibit hesperadin effect and alleviate ferroptosis.

**Conclusion:**

Hesperadin can promote ferroptosis in K562 cells by regulating the SLC7A11/GPX4 axis.

慢性髓性白血病（CML）是一种起源于造血干细胞的恶性增殖性疾病，以Ph染色体和BCR::ABL融合基因为特征[1]。酪氨酸激酶抑制剂（TKI）如伊马替尼被用作一线治疗药物[2]。然而，TKI的原发性和继发性耐药降低了抗白血病的治疗效果。此外许多患者还经历TKI不耐受。铁死亡是一种铁依赖和细胞内氧化积累为特征的程序性细胞死亡[3]。铁死亡失调与肿瘤的发生密切相关，越来越多证据表明铁死亡是抑制肿瘤的重要靶点。近年来发现中药作为天然化合物具有明确的抗肿瘤特性，逐渐成为研究的热点。

橙皮苷（Hesperadin）别名陈皮苷、橘皮苷，是一种广泛存在于柑橘中的天然酚类化合物[4]。最初它被认为是一种抗肿瘤药物，在体外通过抑制AuroraB激酶诱导多种肿瘤细胞的增殖阻滞和凋亡，具有促凋亡、抗氧化、抗炎等功能[5]。然而在CML中作用及机制研究甚少。本研究主要探讨橙皮苷诱导CML细胞发生铁死亡的作用及分子机制。

## 材料与方法

一、材料

1. 细胞：人CML细胞系K562细胞购自武汉普诺赛生命科技有限公司。

2. 试剂与试剂盒：橙皮苷、铁死亡抑制剂（Ferrostatin-1，Fer-1）均为美国MCE公司产品。胎牛血清、RPMI 1640培养基为美国GIBCO公司产品。C11-BODIPY为美国Thermo Fisher公司产品。FerroOrange为日本同仁化学研究所产品。SLCTA11、GPX4抗体购自武汉三鹰生物技术有限公司。Annexin Ⅴ-FITC/PI细胞凋亡检测试剂盒购自赛文创新生物科技有限公司。CCK-8检测试剂盒为日本同仁化学研究所产品，Transwell小室为美国Corning公司产品。EDU试剂盒购自碧云天生物技术有限公司。

二、方法

1. 细胞培养：将CML细胞系K562细胞培养于RPMI 1640培养基（含10％胎牛血清、1％链霉素和1％青霉素）中，并置于含5％CO_2_、37 °C的培养箱中孵育。

2. CCK-8法检测细胞活力：将对数生长期的K562细胞按照2×10^4^/孔的密度接种于96孔培养板中。分别设置空白对照组和不同浓度的橙皮苷组（0.2、0.4、0.6、0.8、1 µmol/L），每组设3个复孔。在24 h以后加入CCK-8试剂（10 µl），在5％CO_2_、37 °C培养箱中孵育2～4 h，使用酶标仪读取450 nm处的吸光度值。

3. EDU试剂盒检测细胞增殖：使用不同浓度的橙皮苷干预K562细胞24 h后，将37 °C预热的2× EDU工作液（20 µmol/L）等体积加入6孔板中，继续孵育2 h，EDU标记完成后加入固定液，室温固定10～15 min。随后加入通透液，室温孵育15 min。经本试剂盒处理后，增殖的细胞在荧光显微镜下呈现出明亮的红色荧光。

4. Transwell检测细胞迁移能力：取K562细胞调整至2×10^4^/ml，加入Transwell小室，24孔板下室加入完全培养基和不同浓度的橙皮苷。经药物干预24 h以后，弃去培养基，用甲醛固定30 min，用PBS清洗3次后，加入0.1％的结晶紫染色30 min。最后用棉签擦去上层未迁移的细胞，在显微镜下观察并拍照。

5. 流式细胞术检测细胞凋亡率：橙皮苷浓度分别为0、0.4、0.8 µmol/L，干预K562细胞24 h后离心收集；使用PBS洗涤3次，加入500 µl的1× Annexin Ⅴ缓冲液悬浮细胞。然后加入5 µl Annexin Ⅴ-FITC混匀后，再加入5 µl Propidium Iodide混匀；室温避光反应5～15 min；在1 h内使用流式细胞仪检测。

6. C11-BODIPY脂质过氧化测定：橙皮苷干预K562细胞24 h后，加入2 µmol/L的C11-BODIPY（581/591），放在37 °C培养箱孵育30 min。用PBS清洗3遍，使用共聚焦显微镜测量荧光强度。

7. FerroOrange细胞内游离铁水平的测定：将K562细胞接种于荧光培养皿中，在37 °C、5％CO_2_培养箱中孵育24 h后，弃去上清液，用无血清培养基洗涤细胞3次。加入浓度为1 µmol/L的FerroOrange工作液，在5％CO_2_、37 °C培养箱中培养30 min。培养后无需清洗直接在荧光显微镜下进行观察。

8. Western blot法检测铁死亡通路相关蛋白：200×*g*离心5 min收集细胞，加入增强型RIPA裂解液裂解细胞。提取细胞总蛋白进行SDS-PAGE凝胶电泳，电转到PVDF膜上。使用含有5％的脱脂奶粉室温封闭2 h。使用TBST清洗后加入一抗，4度摇床过夜。随后用TBST清洗3次，每次10 min。加入对应属性的二抗，室温孵育1 h，再次使用TBST清洗3次，最后使用成像系统检测蛋白表达情况。

9. SLC7A11过表达质粒转染：将K562细胞密度调整至5×10^5^/ml，接种于6孔板中孵育24 h，转染时细胞密度在70％～80％；转染细胞分为2组：空质粒转染（SLC7A11-NC）组和过表达质粒转染（SLC7A11-OE）组；每组设3个复孔，使用Lipofectamine2000作为转染试剂，转染试剂与质粒比例为1∶1混合后；室温孵育10～15 min后加入6孔板中，转染4～6 h换完全培养基，转染72 h后显微镜观察转染效率，收集细胞做后续实验。

10. 实时荧光定量PCR检测铁死亡相关基因mRNA水平变化：TRIzol法提取细胞总RNA，使用逆转录试剂盒将RNA逆转录为cDNA，以NAPDH为内参，使用荧光染料法进行荧光定量PCR，整个感应体系分为扩增和溶解曲线两部分。使用2^−ΔΔCt^表示基因表达水平。引物序列见表1。

**表1:** PCR引物信息

引物名称	引物序列（5′→3′）
SLC7A11-F	TTTGTTGCCCTCTCCTGCTTTG
SLC7A11-R	AGTGTGCTTGCGGACATGAATC
GPX4-F	CCGCTGTGGAAGTGGATGAAG
GPX4-R	TGTCGATGAGGAACTGTGGAGAG
ACSL4-F	TCTGCTTCTGCTGCCCAATT
ACSL4-R	CGCCTTCTTGCCAGTCTTTT
LPCAT3-F	CCTACCTCATCCACCTCTTC
LPCAT3-R	AGTCAACAGCCAAACCAATC
GAPDH-F	GAAGGTGAAGGTCGGAGTC
GAPDH-R	GAAGATGGTGATGGGATTTC

**注** F：正向引物；R：反向引物

三、统计学处理

使用SPSS 24.0软件进行统计分析，数据以均数±标准差表示，所有数据都来自3次独立实验。采用单因素方差分析及*t*检验进行组间比较。*P*<0.05为差异具有统计学意义。

## 结果

一、橙皮苷对K562细胞活力、增殖和迁移的影响

我们首先通过CCK-8检测橙皮苷对K562细胞活力的影响，结果显示：0、0.2、0.4、0.6、0.8、1.0 µmol/L橙皮苷干预组的K562细胞活力分别为（100±0.02）％、（85.50±0.06）％、（60.81±0.01）％、（55.43±0.03）％、（34.20±0.03）％、（22.95±0.02）％。橙皮苷的IC_50_值为0.544 µmol/L。随着橙皮苷浓度的升高，K562细胞活力显著降低。选取0.4 µmol/L（低浓度）、0.8 µmol/L（高浓度）的橙皮苷行后续实验。随后，我们进行了EDU-594细胞增殖和Transwell实验，结果显示：橙皮苷可以抑制细胞增殖（图1A和1B）和迁移（图1C和1D），且呈浓度依赖性。

**图1:**
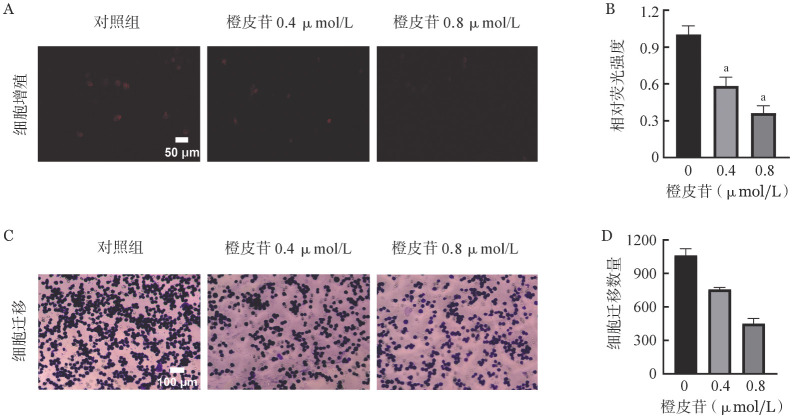
橙皮苷对K562细胞活力、增殖和迁移的影响 **A、B** EDU-594细胞增殖实验检测各组增殖率；**C、D** Transwell检测各组迁移率 **注** 与对照组比较，^a^*P*<0.05

二、橙皮苷对K562细胞凋亡的影响

使用不同浓度橙皮苷干预细胞24 h，从图2A和2B可见低浓度和高浓度组K562细胞凋亡率分别为16.0％、37.4％。进一步采用Western blot法检测凋亡相关蛋白的变化，发现在橙皮苷作用下，抗凋亡蛋白Bcl-2表达下调，促凋亡蛋白Bax及Cleaved-Caspase-3的表达升高（图2C～F）。并且与低浓度相比，高浓度的作用更显著，与图2A流式细胞术检测结果一致。表明橙皮苷可以诱导人CML细胞系K562细胞凋亡。

**图2:**
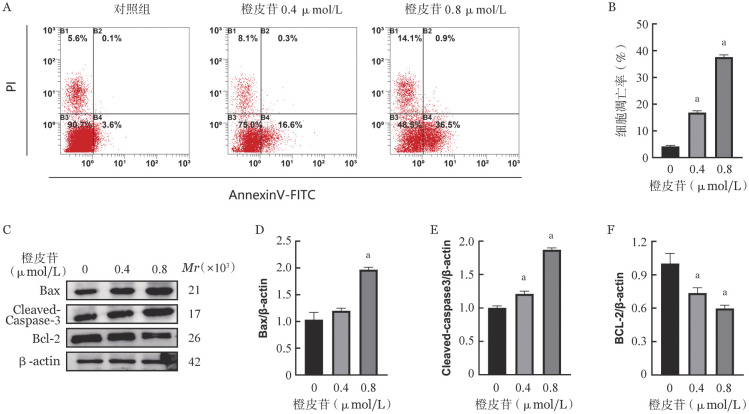
橙皮苷对K562细胞凋亡的影响 **A、B** 流式细胞术检测K562细胞凋亡率；**C～F** Western blot法检测凋亡相关蛋白表达 **注** 与对照组比较，^a^*P*<0.05

三、橙皮苷对K562细胞铁死亡的影响

为了研究橙皮苷是否可以诱导CML细胞铁死亡，我们首先检测了K562细胞内的脂质过氧化（C-11BODIPY）水平。结果显示：与对照组相比，橙皮苷可以显著增加K562细胞内脂质过氧化，且呈剂量依赖性（图3A、3B）。我们进一步使用特异性Fe^2+^探针FerroOrange对细胞内不稳定铁进行了检测，同样发现橙皮苷处理细胞后FerroOrange红色荧光升高，表明Fe^2+^在K562细胞中积累（图3C、3D）。以上结果表明，橙皮苷可促进K562细胞铁死亡。

**图3:**
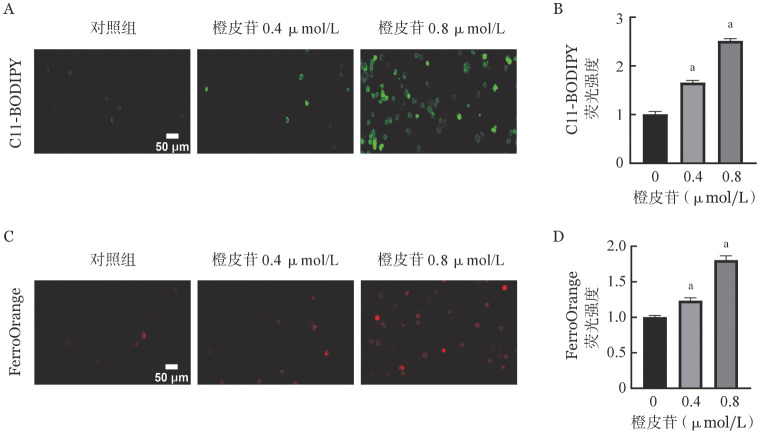
橙皮苷对K562细胞铁死亡的影响 **A、B** C11-BODIPY检测各组细胞内脂质过氧化水平；**C、D** FerroOrange检测铁水平 **注** 与对照组比较，^a^*P*<0.05

四、加入铁死亡抑制剂Fer-1对K562细胞活力、增殖和迁移的影响

为进一步证实铁死亡是否参与橙皮苷介导的抗癌活性，我们引入铁死亡抑制剂Fer-1逆转橙皮苷对K562细胞的抑制作用。CCK-8实验显示：Fer-1+0.4 µmol/L橙皮苷组细胞活力为（81.46±0.06）％，高于0.4 µmol/L橙皮苷组的（57.90±0.05）％，Fer-1+0.8 µmol/L橙皮苷组细胞活力为（52.46±0.07）％，高于0.8 µmol/L橙皮苷组的（35.91±0.04）％（图4A）。EDU-594（图4B、4C）和Transwell（图4D、4E）实验结果显示：与单独橙皮苷组相比，联合铁死亡抑制剂组K562细胞的增殖和迁移率升高。

**图4:**
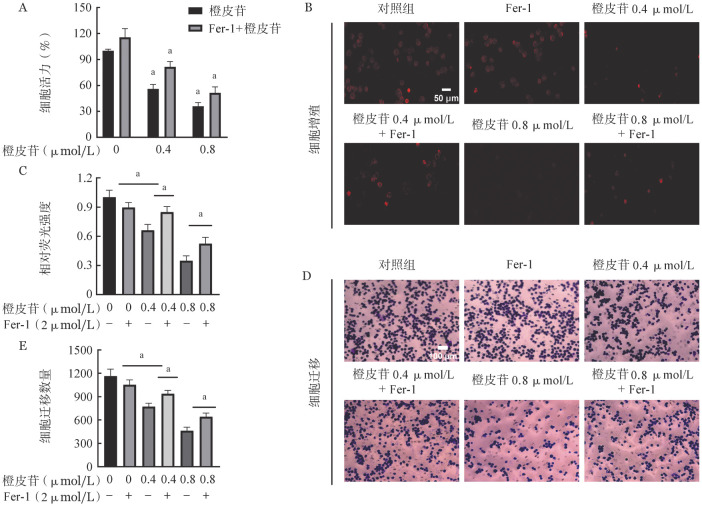
加入铁死亡抑制剂Fer-1检测各组K562细胞活力、增殖、迁移的变化 **A** CCK-8实验检测各组细胞活力；**B、C** EDU-594检测各组细胞增殖率；**D、E** Transwell检测各组迁移率 **注** 与对照组比较，^a^*P*<0.05

五、加入铁死亡抑制剂Fer-1对K562细胞凋亡率的影响

流式细胞术检测结果显示：0.4 µmol/L橙皮苷组细胞凋亡率为17.6％，0.4 µmol/L橙皮苷+Fer-1组细胞凋亡率为9.1％；0.8 µmol/L橙皮苷组细胞凋亡率为41.1％，0.8 µmol/L橙皮苷+Fer-1组细胞凋亡率为29.5％。与单独橙皮苷组相比，联合组细胞凋亡率降低。Fer-1逆转了K562细胞中橙皮苷诱导的细胞死亡。

六、铁死亡抑制剂Fer-1对橙皮苷诱导的K562细胞铁死亡的影响

C11-BODIPY实验结果显示：相比于对照组，橙皮苷组的脂质过氧化水平明显升高，橙皮苷+Fer-1联合组脂质过氧化水平降低（图5A、5B）。同样FerroOrange检测结果显示：橙皮苷在联合使用铁死亡抑制剂Fer-1后，红色荧光强度明显降低（图5C、5D）。这些数据表明，橙皮苷能够诱导K562细胞发生脂质过氧化以及Fe^2+^积累，而铁死亡抑制剂Fer-1可以阻止这一现象。

**图5:**
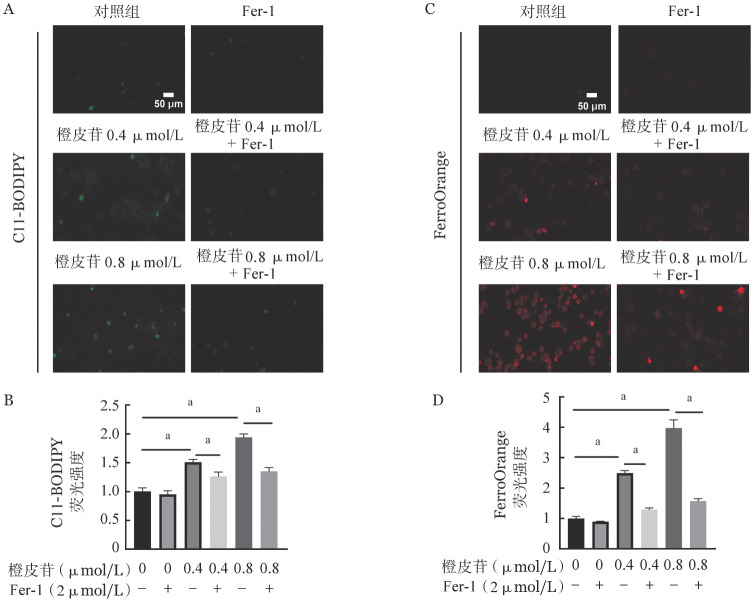
铁死亡抑制剂Fer-1对橙皮苷诱导的K562细胞铁死亡的影响 **A、B** 加入铁死亡抑制剂Fer-1后检测C11-BODIPY变化；**C、D** FerroOrange检测加入铁死亡抑制剂Fer-1后Fe^2+^水平 **注** 两组比较，^a^*P*<0.05

七、橙皮苷通过SLC7A11/GPX4信号轴促进K562细胞铁死亡

为进一步探究橙皮苷调控K562细胞铁死亡的机制，我们选取橙皮苷0.8 µmol/L进行了后续实验。SLC7A11、GPX4、ACSL4、LPCAT3和FTH1被认为是铁死亡发生的关键调控蛋白[6]，实时荧光定量PCR结果显示：与对照组相比，橙皮苷组SLC7A11、GPX4 mRNA水平显著降低，ACSL4、LPCAT3和FTH1无明显变化（图6A）。Western blot结果显示：与对照组相比，橙皮苷组SLC7A11和GPX4蛋白的表达显著降低（图6B～D）。进一步我们在K562细胞中过表达了SLC7A11（图6E、6F），并观察其对橙皮苷作用的影响，结果表明：与对照组相比，橙皮苷组SLC7A11、GPX4蛋白水平下降。脂质过氧化及Fe^2+^水平升高。与SLC7A11-NC+橙皮苷组相比，SLC7A11-OE+橙皮苷组SLC7A11、GPX4蛋白水平升高（图6G～I），脂质过氧化（图6J、6K）及Fe^2+^（图6L、6M）水平降低。以上结果提示SLC7A11/GPX4信号介导了橙皮苷诱导的K562细胞铁死亡。

**图6:**
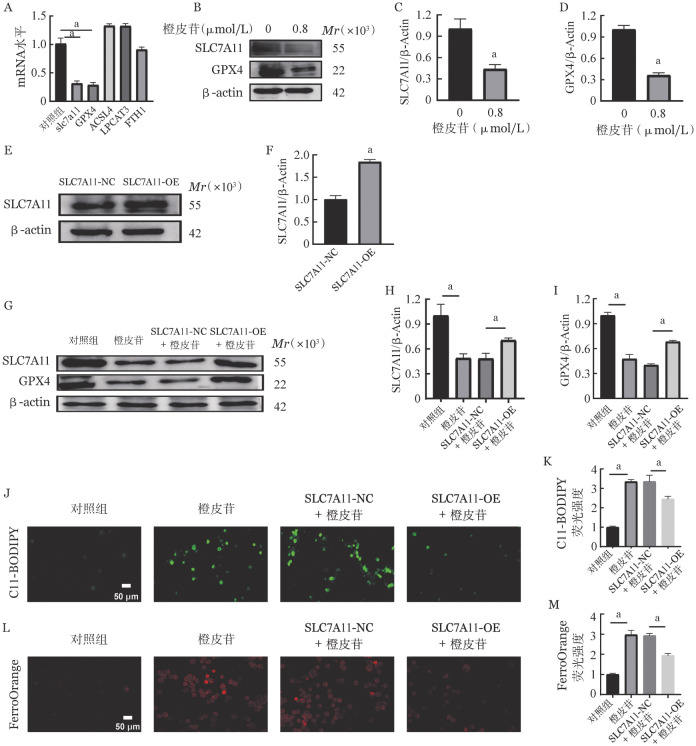
橙皮苷通过SLC7A11/GPX4信号轴促进K562细胞铁死亡 **A** 橙皮苷浓度为0.8 µmol/L时SLC7A11、GPX4、ACSL4、LPCAT3 mRNA水平；**B～D** Western blot检测橙皮苷作用后K562细胞SLC7A11、GPX4蛋白水平变化；**E、F** 过表达SLC7A11；**G～I** Western blot检测过表达SLC7A11 K562细胞橙皮苷作用后SLC7A11、GPX4的蛋白表达量；**J、K** C11-BODIPY检测各组脂质过氧化水平；**L、M** FerroOrange检测各组铁离子水平 **注** 两组比较，^a^*P*<0.05

## 讨论

伊马替尼作为第一代BCR::ABL酪氨酸激酶抑制剂，广泛用于CML的一线治疗，但是部分患者对伊马替尼耐药[7]已成为临床治疗的主要问题。在本研究中我们发现，铁死亡在橙皮苷诱导的K562细胞死亡方式中占有主要地位。铁死亡是一种新形式的程序性细胞死亡，不同于细胞凋亡、坏死和自噬等形式。其特征是脂质过氧化物和铁离子的积累[8]。铁是驱动细胞内脂质过氧化和铁死亡的重要物质[9]。发生铁死亡时，细胞内的胱氨酸摄取和谷胱甘肽（GSH）的合成减少，最终导致细胞死亡[10]。在过去的十年中，铁死亡被发现与多种肿瘤的发生发展和治疗相关，并作为肿瘤的抑制机制发挥作用。因此，靶向铁死亡为癌症治疗提供了一个很有前途的策略[11]。

本研究结果显示橙皮苷的活性是通过促进铁死亡发挥作用。越来越多的证据表明橙皮苷具有抗肿瘤作用。它能抑制细胞增殖、迁移以及诱导细胞周期阻滞和凋亡。凋亡和铁死亡是两种类型的程序性细胞死亡，但是彼此密切相关，铁死亡可以促进细胞对凋亡的敏感性[12]。铁死亡和凋亡途径是潜在的癌症治疗策略，据报道许多药物通过组合途径发挥抗癌活性[13]–[15]。在本研究中，橙皮苷通过SLC7A11/GPX4通路促进细胞发生铁死亡，并且可能通过铁死亡和细胞凋亡的混合途径发挥抗癌活性。在其他疾病中，橙皮苷通过IL-6/STAT3信号通路，抑制肺成纤维细胞衰老，阻碍肺纤维化的进展[16]。橙皮苷通过TGF-β1/Smad信号通路抑制细胞增殖，可以改善良性前列腺增生[17]。橙皮苷通过NRF2/NF-κB轴减轻髓核细胞中氧化应激诱导铁死亡，避免椎间盘退化[18]。在肺癌细胞中橙皮苷可以通过靶向miR-132/ZEB2信号通路促进细胞凋亡，抑制增殖[19]。橙皮苷还可以通过抑制SDF-1/CXCR-4通路抑制非小细胞肺癌的迁移和侵袭[20]。在膀胱癌中，通过PI3K/AKT/FOXO3a通路促进细胞凋亡和周期阻滞[21]。本研究以0.2、0.4、0.6、0.8、1.0 µmol/L橙皮苷处理K562细胞，均可降低K562细胞活力；采用0.4、0.8 µmol/L橙皮苷处理K562细胞，可降低其细胞增殖和迁移率。流式细胞术发现橙皮苷可以明显诱导K562细胞凋亡。Western blot法检测显示，橙皮苷可下调K562细胞内BCL-2的表达，并上调Cleaved-caspase 3和Bax的表达水平，这一结果表明橙皮苷可增加促凋亡蛋白，同时下调抑凋亡蛋白的表达，诱发K562细胞凋亡，减弱其增殖活性。因此，橙皮苷可发挥显著的抗癌功效；剂量越高，功效越强。我们推测橙皮苷在治疗CML中，是一种很有前途的药物。

SLC7A11是一种多通道跨膜蛋白，可调节XC-系统（含有SLC7A11和SLC3A2亚单位的跨膜蛋白复合物）中的胱氨酸/谷氨酸逆向转运蛋白活性[22]。越来越多的证据证明，SLC7A11可以抑制铁死亡，促进肿瘤的进展。例如在肿瘤抑制过程中，p53通过抑制SLC7A11的表达使细胞对铁死亡敏感[23]。心脏铁蛋白的缺失通过SLC7A11介导的铁死亡促进心肌病的形成[24]。SLC7A11抑制时，肿瘤抑制因子BRCA1相关蛋白1（BAP1）会引发铁死亡，从而减缓肿瘤进展[25]。RNA结合蛋白（RBP）对SLC7A11翻译后调节，促进肺癌中的铁死亡[26]。HBV X蛋白（HBV X protein，HBx）通过组蛋白甲基转移酶（EZH2）抑制SLC7A11，促进急性肝衰竭发生铁死亡[27]。此外在胃癌[28]、肺癌[29]等肿瘤细胞中SLC7A11是高表达的，在抑制SLC7A11后可诱发细胞死亡。黄芩苷通过NRF2/SLC7A11/GPX4调节轴诱导骨肉瘤发生铁死亡[30]。辣椒素通过体外调节SLC7A11/GPX4信号轴诱导NSCLC细胞铁死亡[31]。Ruscogenin通过NRF2/SLC7A11/GPX4信号通路抑制软骨细胞铁死亡，减轻骨关节炎中的软骨破坏[32]。青蒿琥酯通过SLC7A11/GPX4介导的铁死亡抑制胰岛素瘤细胞的生长[33]。二甲双胍通过抑制乳腺癌中SLC7A11诱导铁死亡[34]。因而推测橙皮苷可能通过抑制SLC7A11/GPX4轴，促进K562细胞铁死亡。本研究结果显示，以0.4、0.8 µmol/L橙皮苷处理K562细胞，可升高细胞内脂质过氧化和铁水平。而加入铁死亡抑制剂Fer-1后各项指标水平得到逆转，0.8 µmol/L橙皮苷作用K562细胞24 h后，有效降低细胞内SLC7A11和GPX4蛋白的表达。这表明橙皮苷可以促进铁死亡。评估SLC7A11在K562细胞中的功能，进一步使用SLC7A11-OE转染K562细胞，对SLC7A11进行过表达。Westen blot检测显示，与单独使用橙皮苷组相比，SLC7A11-OE+橙皮苷组可降低细胞内脂质过氧化和铁离子水平。以上结果证明：过表达SLC7A11后，可以减弱橙皮苷对K562细胞的杀伤作用。

综上所述，本研究证实了橙皮苷可以抑制K562细胞增殖，通过下调SLC7A11/GPX4通路蛋白表达，引发K562细胞内脂质过氧化和铁累积，诱导细胞铁死亡，这可能是橙皮苷抗CML细胞的作用机制之一。橙皮苷下调SLC7A11/GPX4信号通路的具体分子机制还待深入研究。
